# Genotypic and phenotypic analysis of biofilm formation *Staphylococcus epidermidis* isolates from clinical specimens

**DOI:** 10.1186/s13104-020-04965-y

**Published:** 2020-02-27

**Authors:** Bahman Mirzaei, Peyman Faridifar, Mostafa Shahmoradi, Reza Shapouri, Fatemeh Iranpour, Fakhri Haghi, Mahnaz Ezzedin, Reyhaneh Babaei, Seyed Fazlollah Mousavi

**Affiliations:** 1 Department of Microbiology and Virology, School of Medicine, Mazandaran University of Medical Sciences, Sari, Mazandaran Iran; 2 Department of Microbiology, Islamic Azad University, Zanjan Branch, Zanjan, Iran; 3 Department of Microbiology, Islamic Azad University, Tehran Branch, Tehran, Iran; 4 Department of Microbiology and Virology, Faculty of Medicine, Zanjan University of Medical Sciences, Zanjan, Iran; 5 Department of Microbiology, Microbial Research Center, Pasteur Institute of Iran, Tehran, Iran

**Keywords:** *Staphylococcus epidermidis*, Biofilm-related genes, Multiplex colony PCR, *atlE*, *sdrG*, *icaADCB*

## Abstract

**Objectives:**

*Staphylococcus epidermidis* is the primary causative agent of infections associated with indwelling biomaterials. Antibiotic susceptibility patterns, Biofilm formation capability, and screening of responsible genes in biofilm formation procedure in clinical isolates (*icaA*, *icaB, icaC*, *icaD*, *sdrG,* and *atlE*) were assigned as the main objectives in this study. The clinical samples were analyzed via standard biochemical assays for identifying different bacteria which were confirmed using the multiplex colony PCR method. Subsequently, biofilm-formation capability, antibiotic susceptibility testing, and the frequency of genes responsible for biofilm formation in the confirmed strains were checked.

**Results:**

Out of 183 clinical specimens 54 *S. epidermidis* isolates were detected by targeting a housekeeping gene (*sesc*) taking advantage of the PCR procedure. All of the strains were Biofilm forming producers. The in vitro biofilm formation assays determined that 45 (83.33%), 5 (9.26%), 4 (7.41%) were strong, moderate, and weak biofilm former strains respectively. Among the isolated strains, the specific frequencies of the biofilm-forming genes were specified to be (98%) for *sdrG*, (84%) for *atlE*, (80%) for *icaC*, and (70%) for *icaD*. Cefamandole and Amikacin are the most effective antibiotics in isolated strains. All strains were ascertained to be methicillin and amoxicillin/clavulanic acid resistant.

## Introduction

Among the CNS, *S. epidermidis* is the most frequently isolated species accounting for three- quarters of the CNS recovered from clinical specimens and approximately 75% of CNS infections [1]. *S. epidermidis* was ranked second in surgical site infections. Moreover, because of biofilm-forming capability as the main virulence factor this bacterium implicated in 34% of cases in catheter-related infections (CRIs) in children and 51% in neonates [2, 3]. The recent findings in Iran were shown *S. epidermidis* isolated strains from various clinical specimens more ubiquitous prevalence in other Iranian cities (73.9%) than Tehran (56.65%) [4]. Other worldwide conducted researches also have shown high isolation rates of the mentioned bacterium in clinical specimens, especially in medical devices samples [5–7]. Biofilms are notoriously resistant to immune system responses and antimicrobial agents. They are a huge problem in industry and medicine (responsible for ≈ 65% of all bacterial infections) [8]. Biofilm formation in *S. epidermidis* was completed by factors including surface proteins such as staphylococcal surface proteins (Ssp-1, Ssp-2), Bap homolog protein (Bhp), autolysin E (AtlE) such as (fibrinogen-binding protein of *S. epidermidis*) SdrG (Fbe), respectively, and likewise by generating polysaccharide intracellular adhesion by *ica* Luci [9]. Initial attachment is accomplished by staphylococcal surface proteins e.g. Ssp-1, Ssp-2, bap homolog protein (Bhp), autolysin E (AtlE), and teichoic acids [9]. Indirect binding to the surface of implanted medical devices is done through microbial surface components recognizing adhesive matrix molecules (MSCRAMMs) such as fibrinogen-binding protein of *S. epidermidis* (SdrG or Fbe), extracellular matrix binding protein (Embp), *S. epidermidis* lipase protein (GehD), AtlE, and elastin-binding protein of *S. aureus* (Ebps) [9]. In Staphylococci, the production of polysaccharide intercellular adhesion [PIA, also called poly-*N*-acetyl glucosamine (PNAG)] by the genes in the intercellular adhesion (*ica*) operon is the best-understood mechanism [10]. On account of the undeniable role of biofilm formation in *S. epidermidis* pathogenesis, the prevalence of the mentioned bacterium in clinical specimens and screening of biofilm-related genes in biofilm-forming strains, and their antibiotic susceptibility patterns are main objectives in this study.

## Main text

### Materials and methods

#### Isolation and Identification of Staphylococci

A total of 200 clinical specimens including urine, blood, sputum, CSF, pleura, fluid, and wound from three educational hospitals (Sina, Imam Khomeini, and Shariati) were collected from different clinical samples of patients suspected to have a clinical infection from October 2015 to March 2016. Identification was done according to Bergey’s Manual of Determinative Bacteriology guideline [11]. Furthermore, following 24–48 h of incubation at 37 °C on Blood agar, using conventional microbial tests including Gram staining, Capability of mannitol fermentation, production of coagulase enzyme, and susceptibility to novobiocin antibiotic disc were done to differentiate isolates.

#### Reconfirmation of *S. epidermidis* isolates

Molecular identification of *S. epidermidis* was performed by targeting the *sesc* gene encoding a surface protein specific for *S. epidermidis* using specifically designed primers which produced a 388 bp PCR product [12]. Amplification was done in a total volume of 25 μl containing 14 μl master amplicon (Biolab, New England, UK), 1 pmol of each forward and reverse primers, a minor amount of colony as a template and 9 μl distilled water. The first cycle of denaturation was at 95 °C for 5 min, followed by 25 cycles at 95 °C for 30 s, then at 55 °C for 1 min, at 72 °C for 1 min, and finally a terminal extension for 5 min. Additional file 1.

#### Antibiotic susceptibility testing

Susceptibility testing was performed according to the CLSI standard guidelines [13] using the disks (BD BBLTM Sensi DiscTM) containing penicillin 2 µg, amikacin 10 µg, chloramphenicol 30 µg, ciprofloxacin 5 µg, levofloxacin 5 µg, gentamycin 120 µg, kanamycin 30 µg, erythromycin 15 µg, ceftriaxone 30 µg, ceftazidime 30 µg, cefotaxime 30 µg, and cefoxitin 30 µg.

#### In vitro biofilm formation assay

Biofilm formation capability was accomplished as previously described [14]. Briefly, the optical density of inoculated colonies on the trypticase soy broth was adjusted to the 0.7 in 600 nm. Afterward, 200 μl of 1:200 diluted (OD 0.005 in 600 nm) suspension in trypticase soy broth supplemented with 1% glucose (TSBg) was transferred into a polystyrene microtiter plate (Nunc, Roskilde, Denmark). Following the incubation at 37 °C for 16 h, supernatants and planktonic cells were removed by three times washing with phosphate-buffered saline (PBS). The wells were stained with 150 μl of 0.1% crystal violet and the additional stain was removed by two-times washing with PBS buffer. The optical density of resolved crystal violet in 160 μl alcohol/acetic acid solution (4:1 concentration) was measured at 595 nm. Each sample was tested in triplicate and the ability of biofilm-forming was measured using the following formula. Optical density cut-off value (ODc) = average OD of negative control + 3 × standard deviation (SD) of negative control.

#### Determining the biofilm-related genes by multiplex colony PCR

In the presence of genes encoding intracellular adhesion (*icaA, icaB, icaC, icaD*), *sdrG*, and *atlE* genes were assessed as previously demonstrated [15]. PCR was performed with a total volume of 50 μl contained 30 μl of master amplicon (Biolab, New England, UK), 1 pmol of each primer, and a minor amount of fresh colony as a template using Gene Amp PCR system (Applied Biosystems, USA). Multiplex colony PCR was carried out in two series: the first series was optimized for the understanding of *ica* locus genes and the other series were optimized for understanding of sdrG with total volume of 50 μl, containing 30 μl master amplicon (Biolab, New England, UK), 2 pmol of each forward and reverse primers, a negligible amount of colony as the template, and 14 μl distilled water. In the actual utilization of program, the initial cycle of denaturation was carried out at 95 °C for 2 min, followed by 50 cycles at 95 °C for 30 s, then at 54 °C for 1 min (55 °C for *sdrG* and *atlE*), at 72 °C for 1 min, 1 min for the final extension, and 7 min for the terminal extension. Resulting PCR was visualized with 3% agarose gel (KBC, Max Pure agarose, Spain). Sequences of utilized primers are detailed in Additional file 2.

### Results

Out of 183 staphylococcal isolates recovered from clinical samples, 80 (43.72%) isolates were identified as *S. epidermidis* using the biochemical procedure. By targeting housekeeping *S. epidermidis* sequence (sesc gene) out of 80 isolates 54 (29.51%) strains were precisely reconfirmed.

The highest and lowest isolation rates belong to Blood (70%) and bone marrow (0%). Detailed data to *S. epidermidis* isolates according to the clinical sample sources are specified in Table 1.

**Table 1:** Frequency of *S. epidermidis* isolates according to clinical samples

Clinical samples		Identified *S. epidermidis* by biochemical tests	Identified *S. epidermidis* by PCR
Sample	N (%)	N (%)	N (%)
Blood	95 (51.9%)	53 (67.9%)	37 (74%)
Trachea	39 (21.3%)	11 (14.4%)	6 (12%)
Urine	15 (8.19%)	4 (5.1%)	3 (6%)
Wound	16 (8.74%)	5 (6.4%)	1 (2%)
Pleura	9 (4.91%)	2 (2.5%)	1 (2%)
Catheter	6 (3.27%)	2 (2.5%)	4 (7.40%)
Bone marrow	1 (0.54%)	1 (1.2%)	0 (0%)
Eye discharge	2 (1.09%)	2 (2.5%)	3 (4%)
Total	183 (100%)	80 (100%)	54 (100%)

As observed in Additional file 3, the most effective antibiotics were cefamandole (93%) and amikacin 64.4%. Moreover, resistance to penicillin 100%, cefoxitin 95.6%, amoxicillin/clavulanic Acid 88.9% were respectively.

Among all 54 slime producing *S. epidermidis* strains tested for biofilm formation by quantitative method, 45 (83.33%), 5 (8.26%), and 4 (7.41%) strains exhibited high ($4{\text{OD}}_{\text{C}} < OD$), moderate (${\text{OD}}_{\text{C}} < OD < 2O{\text{D}}_{\text{C}}$), and weak (${\text{OD}}_{\text{C}} < OD < 2O{\text{D}}_{\text{C}}$) levels of biofilm formation, respectively. Tables 2, 3.

**Table 2:** Frequency of biofilm-related genes in *S*. *epidermidis* isolates

Biofilm related genes	Biofilm forming capacity	
	Moderate (n = 5)	Strong (n = 45)
*icaA*^+^	1 (20%)	23 (51.1%)
*icaA*^−^	4 (80%)	22 (48.9%)
*icaB*^+^	2 (40%)	5 (11.11%)
*icaB*^−^	3 (60%)	40 (88.88%)
*icaC*^+^	3 (60%)	37 (82.8%)
*icaC*^−^	2 (40%)	8 (18.8%)
*ica D*^+^	3 (60%)	32 (71.1%)
*ica D*^−^	2 (40%)	13 (28.9%)
*icaADCB*^+^	1 (20%)	23 (51.1%)
*icaADCB*^−^	4 (80%)	22 (48.9%)
*sdrG*^+^	3 (60%)	39 (86.7%)
*sdrG*^−^	2 (40%)	6 (13.31%)
*atlE*^+^	4 (80%)	45 (100%)
*atlE*^−^	1 (20%)	0 (0%)
*sdrG, atlE*^+^	3 (60%)	39 (86.7%)
*sdrG, atlE*^−^	2 (40%)	6 (13.31%)

**Table 3:** Patterns of biofilm-related genes in biofilm-forming isolates

Biofilm related genes		Prevalence of biofilm related genes (N)	Number of isolates with presence of genes (N)
Genes patterns	Number of genes (N)		
*icaADBC, sdrG and atlE*	6	42	21
*atlE, sdrG, icaD,B,C*	5	8	4
*atlE, icaADBC*	5	8	4
*icaA,D,B,C*	4	16	8
*atlE, icaD,B,C*	4	16	8
*atlE, sdrG, icaD, icaC*	4	16	8
*icaD, sdrG, atlE*	3	12	6
*icaC, atlE, sdrG*	3	12	6

It seems that more research is needed for determining the role of genes product in *S. epidermidis* strains and finding the relationship between the expression of product and biofilm-formation procedure in mentioned bacteria. In the current study, only the presence of genes responsible for biofilm formation process has been assessed. Expression of the related genes product to the evaluation of their role in the biofilm-formation procedure has not been performed yet

While screening for the genes associated with attachment to the host cells, *sdrG*, *atlE, icaC, and icaD* (98%, 84%, 80%, and 70%, respectively) were determined to be the predominant genes found within the clinical isolates. These genes were found to occur at a much higher frequency than the *icaB* and *icaA* genes (54% and 48%, respectively). Among the strong biofilm-forming isolates, the frequency of *fnbA* and *cna* genes were higher than in those of the moderate biofilm-forming strains. Considering the Chi square exact (Fisher exact for *atlE*) meaningful difference between strong and moderate biofilm producer strains related genes was not observed. Additional files 3, 4.

### Discussion

Due to the ubiquitous prevalence of *S. epidermidis* as a commensal bacterium, it is often difficult for a clinician to decide whether an isolate represents the causative agent of infection or unspecific culture contamination [16]. Considering a systematic review study in Iran isolation rates of *S. epidermidis* strains in Tehran were reported 99 (2011), 64 (2014), and 20 (2015) [4]. The mentioned study reported that *S. epidermidis* isolates more prevalent in Tabriz, Arak, Sanandaj than in Tehran. At the current research, the prevalence of *S. epidermidis* was estimated as 54 (29.51%). Regarding the number of isolates in Tehran, Similar results were observed in the previously published studies. The frequency of *S. epidermidis* in other studies in Belgium 2013 (33), Africa 2018 (20), and Netherland 2019 (27) were assessed respectively [5–7]. Biofilm formation capability could enhance the durability of the mentioned bacterium against disinfection principles in hospitals in the current study [16]. In this research, various capabilities of biofilm formation of *S*. *epidermidis* isolates were observed. A baseline has been calculated on three standard deviations: weak, moderate and strong biofilm formers. In this way, merely five (5%) strains out of 50 were mild-biofilm formers (2 × ODc < OD < 4 × ODc) and Forty-five (90%) isolates could be classified as strong biofilm formers (4 × ODc < OD). Observed results happen to be the same as Delpozo and Patel Filho’s findings [8, 17].

In 2016, two different studies investigated clinical isolates of *S. epidermidis* collected from a private hospital in Tehran [18] and clinical isolates from Shiraz city [19]. Zalipour reported that in 81.9% of clinical isolates of *S. epidermidis*, *ica* Luci was observed [19]. Rahimi reported that 82% of the isolated strains were biofilm-formers and all of the biofilm former *S. epidermidis* isolates carried *icaA*, *icaD*, *aap*, and *atlE* genes [18]. Granting to the previous findings, [20] *sdrG* and certain genes have a crucial role in biofilm formation of clinically isolated strains. Our experiments proved and reconfirmed that the mentioned genes have a fundamental role in biofilm formation [20]. Generated PCR products have shown that proteins dependent biofilm formation process is the predominant factor in our isolates. The frequency of generating targeted genes in *ica* Luci was relatively similar to Dimond’s study [21]. According to the already presented data in a previously published study [15], when the gene profile of clinical isolates of *S*. *epidermidis* from orthopedic prosthesis was compared with the phenotypic biofilm-formation ability, 41% of non-biofilm-forming isolates were complete *ica* negative and only 2% harbored all *ica* genes. Our findings represented that all 50 *S*. *epidermidis* isolates were assigned as biofilm-forming strains. The frequency of determining *ica* Luci in strong biofilm-forming strains was 51.1% and also 86.7% for both *atlE* and *sdrG* genes. Our findings showed that *ica* Luci, *sdrG*, and *atlE* genes could improve the ability of biofilm formation in strong status. Complete or relative resistance to β-lactams is a characteristic feature of the genus staphylococci [14]. An extra problem with staphylococci is that they are typically tolerant of β-lactams (i.e., the MBC/MIC of > 32). The major mechanism underlying this resistance has been documented by the production of low-affinity PBP [14]. Bactericidal antibiotics (e.g., ampicillin or penicillin G), for susceptible strains to penicillin and a glycopeptide (e.g., vancomycin) to which the Staphylococci isolate does not exhibit high-level resistance, are the best drugs of choice for staphylococci infections [22]. Our findings show imipenem could be a proper drug of choice to eradicate bacterial infections caused by *S. epidermidis* strain. Vancomycin in combination with an aminoglycoside has demonstrated synergistic activity against staphylococci both in vitro and in vivo [22]. However, staphylococci are becoming increasingly resistant to traditional antibiotic therapy. Besides, high-level aminoglycoside resistance and rapid spread of vancomycin resistance have resulted in limited therapeutic alternatives. Our isolated strains showed that the probable 29% and 31% isolates were resistant to gentamycin and vancomycin, respectively. Susceptibility patterns indicated that cefamandole is the best choice of medication for the treatment of *S*. *epidermidis* related infections [22]. In conclusion, biofilm-forming S. *epidermidis* strains owing to the increasing prevalence of antibiotic resistance to β-lactams and aminoglycoside and consumedly usage of antibiotics must be distinguished by the prompt examination method to avoid medical devices related to infections. Hasty identification of affected genes that encoded proteins and polysaccharides in the construction of biofilm has a considerable influence on the multiplex PCR method.

### Limitation

The patterns of antibiotic resistance did not show a significant correlation with the biofilm formation and related analyzed strains. It seems that more research is needed for determining the role of genes product in *S. epidermidis* biofilm formation.

#### Statistical analysis

In this study, we are using a Chi squared test, (χ^2^ test). Statistical analysis of results was accomplished by using SPSS version 19 and a *P* value < 0.05 was considered significant.

## Supplementary information

**Additional file 1.** Standardization of molecular test (PCR). Generated 388 bp sesc gene by PCR to *S. epidermidis* identification by 1% agarose gel (A). Standardization of biofilm-related genes *ica* Luci: *icaA* (103 bp), *icaB* (302 bp), *icaC* (400 bp), *icaD* (193 bp), *sdrG* (495 bp), and *atlE* (682 bp) by Multiplex colony PCR based on the size in 3% agarose gel (B). M; 100 bp DNA ladder, Lane 1-4; standardization of PCR for each targeted gene by colony PCR, Lane 5; standardization of semiplex PCR (two target genes), Lane 6; Multiplex colony PCR for *ica* Luci genes, Lane 7; optimization of Multiplex colony PCR for *sdrG* and *atlE* genes. Resulting PCR was visualized in a 1% agarose gel. (KBC, Max Pure agarose, Spain). *S. epidermidis* ATCC 12228 and *Escherichia coli* ATCC 25922 were used as the positive and negative control strains.

**Additional file 2.** Sequences of utilized primers in this study.

**Additional file 3.** Antibiotic resistance profile to biofilm-forming isolates. S; Susceptible, R; Resistant, N; the total number.

**Additional file 4.** Frequency of antibiotic resistance pattern for MDR biofilm-forming S. *epidermidis* strains according to the utilized antibiotic. MA; Cefamandole, CTX; Cefotaxime, P; Penicillin, S; Streptomycin, AN; Amikacin, NB; Novobiocin, FOX; Cefoxitin, AMC; Amoxicillin/Clavulanic Acid, K; Kanamycin, CEC; Cefaclor.

## Data Availability

All the results of this study have been classified and maintained by the dissertation in the Pasteur Institute of Iran. We have indeed provided all raw data on which our study is based.
