# Supplementary figures and images for: Genotypic and phenotypic analysis of biofilm formation Staphylococcus epidermidis isolates from clinical specimens
Source: BMC Res Notes. 2020 Feb 27;13:114. doi: 10.1186/s13104-020-04965-y (PMC7045379; doi:10.1186/s13104-020-04965-y)

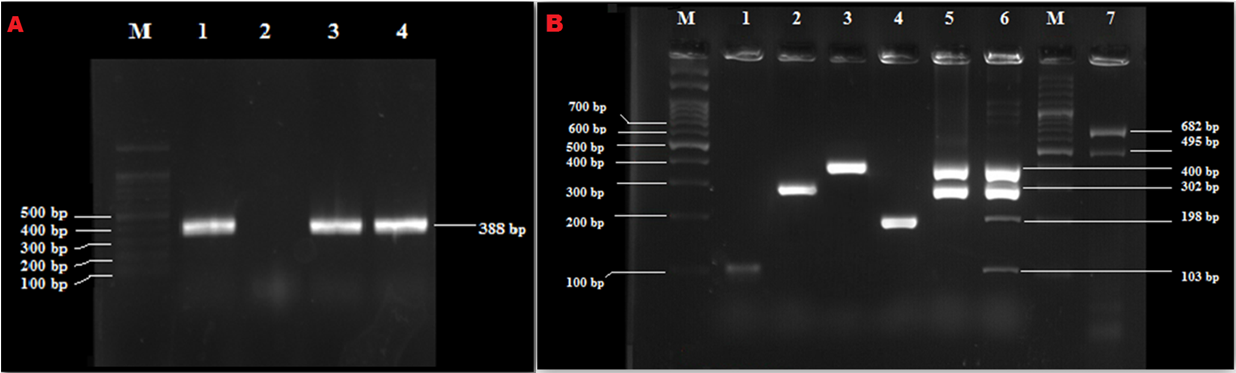

Supplement: Supplementary file 1 — Additional file 1. Standardization of molecular test (PCR). Generated 388 bp sesc gene by PCR to S. epidermidis identification by 1% agarose gel (A). Standardization of biofilm-related genes ica Luci: icaA (103 bp), icaB (302 bp), icaC (400 bp), icaD (193 bp), sdrG (495 bp), and atlE (682 bp) by Multiplex colony PCR based on the size in 3% agarose gel (B). M; 100 bp DNA ladder, Lane 1-4; standardization of PCR for each targeted gene by colony PCR, Lane 5; standardization of semiplex PCR (two target genes), Lane 6; Multiplex colony PCR for ica Luci genes, Lane 7; optimization of Multiplex colony PCR for sdrG and atlE genes. Resulting PCR was visualized in a 1% agarose gel. (KBC, Max Pure agarose, Spain). S. epidermidis ATCC 12228 and Escherichia coli ATCC 25922 were used as the positive and negative control strains. [file 13104_2020_4965_MOESM1_ESM.tif]
