# Supplementary material for: Genotypic and phenotypic analysis of biofilm formation Staphylococcus epidermidis isolates from clinical specimens
Source: BMC Res Notes. 2020 Feb 27;13:114. doi: 10.1186/s13104-020-04965-y (PMC7045379; doi:10.1186/s13104-020-04965-y)
Supplement: Supplementary file 2 — Additional file 2. Sequences of utilized primers in this study. [file 13104_2020_4965_MOESM2_ESM.docx]

| Gene | Primer sequence | Size (bp) |
| --- | --- | --- |
| *icaA* | F 5´-ACA GTC GCT ACG AAA AGA AA-3´  R 5´-GGA AAT GCC ATA ATG ACA AC-3´ | 103 |
| *icaD* | F 5´-ATG GTC AAG CCC AGA CAG AG-3´  R 5´-CGT GTT TTC AAC ATT TAA TGC AA-3´ | 198 |
| *icaB* | F 5´-CTG ATC AAG AAT TTA AAT CAC AAA-3´  R 5´-AAA GTC CCA TAA GCC TGT TT-3´ | 302 |
| *icaC* | F 5´-TAA CTT TAG GCG CAT ATG TTT T-3´  R 5´-TTC CAG TTA GGC TGG TAT TG-3´ | 400 |
| *atlE* | F 5´-CAA CTG CTC AAC CGA GAA CA-3´  R 5´-TTT GTA GAT GTT GTG CCC CA-3´ | 682 |
| *sdrG* | F 5´-TAA ACA CCG ACG ATA ATA ACC AAA-3´  R 5´-GGT CTA GCC TTA TTT TCA TAT TCA-3´ | 495 |
| *sesC* | F 5´-GTT GAT AAC CGT CAA CAA GG-3´  R 5´-CAT GTT GAT CTT TTG AAT CCC-3´ | 388 |
