# Supplementary material for: Genotypic and phenotypic analysis of biofilm formation Staphylococcus epidermidis isolates from clinical specimens
Source: BMC Res Notes. 2020 Feb 27;13:114. doi: 10.1186/s13104-020-04965-y (PMC7045379; doi:10.1186/s13104-020-04965-y)
Supplement: Supplementary file 3 — Additional file 3. Antibiotic resistance profile to biofilm-forming isolates. S; Susceptible, R; Resistant, N; the total number. [file 13104_2020_4965_MOESM3_ESM.docx]

| **Antibiotics** | | **Biofilm forming isolates (N= 50)** | |
| --- | --- | --- | --- |
|  |  | **Moderate (5)** | **Strong (45)** |
| **Cefamandole** | **S** | 4 (80%) | 42 (93.3%) |
|  | **R** | 1 (20%) | 3 (6.7%) |
| **Cefotaxime** | **S** | 2 (40%) | 18 (40%) |
|  | **R** | 3 (60%) | 27 (60%) |
| **Streptomycin** | **S** | 4 (80%) | 24 (53.3%) |
|  | **R** | 1 (20%) | 21 (46.7%) |
| **Penicillin** | **S** | 0 (0%) | 0 (0%) |
|  | **R** | 5 (100%) | 45 (100%) |
| **Amikacin** | **S** | 4 (80%) | 29 (64.4%) |
|  | **R** | 1 (20%) | 16 (35.6) |
| **Cefoxitin** | **S** | 1 (20%) | 2 (4.4%) |
|  | **R** | 4 (80%) | 43 (95.6%) |
| **Amoxicillin**  **/Clavulanic Acid** | **S** | 0 (0%) | 5 (11.1%) |
|  | **R** | 5 (100%) | 40 (88.9%) |
| **Kanamycin** | **S** | 3 (60%) | 20 (44.4%) |
|  | **R** | 2 (40%) | 25 (55.6%) |
| **Cefaclor** | **S** | 4 (80%) | 26 (57.8%) |
|  | **R** | 1 (20%) | 19 (42.4%) |

S; Susceptible, R; Resistant, N; total number.
