# Supplementary material for: Genotypic and phenotypic analysis of biofilm formation Staphylococcus epidermidis isolates from clinical specimens
Source: BMC Res Notes. 2020 Feb 27;13:114. doi: 10.1186/s13104-020-04965-y (PMC7045379; doi:10.1186/s13104-020-04965-y)
Supplement: Supplementary file 4 — Additional file 4. Frequency of antibiotic resistance pattern for MDR biofilm-forming S. epidermidis strains according to the utilized antibiotic. MA; Cefamandole, CTX; Cefotaxime, P; Penicillin, S; Streptomycin, AN; Amikacin, NB; Novobiocin, FOX; Cefoxitin, AMC; Amoxicillin/Clavulanic Acid, K; Kanamycin, CEC; Cefaclor. [file 13104_2020_4965_MOESM4_ESM.docx]

| **Resistance to** | **Antibiotics** | **Number of isolates** |
| --- | --- | --- |
| **Two antibiotics** | P,AMC | 8 |
| **Three antibiotics** | P,AMC,K | 28 |
|  | CTX,P,AMC |  |
|  | P,FOX,AMC |  |
|  | P,S,AN |  |
| **Four antibiotics** | CTX,P,AMC,K | 14 |
|  | CTX,P,FOX,AMC |  |
| **Five antibiotics** | CTX,P,AMC,K | 4 |
|  | CTX,P,FOX,AMC |  |
| **Six antibiotics** | CTX,P,FOX,AMC,K,CEC | 10 |
| **Seven antibiotics** | CTX,P,AN,FOX,AMC,K,CEC | 24 |
|  | MA,CTX,P,FOX,AMC,K,CEC |  |
| **Eight antibiotics** | CTX,P,S,AN,FOX,AMC,K,CEC | 2 |
| **Nine antibiotics** | MA,CTX,P,S,AN,FOX,AMC,K,CEC | 4 |

MA; Cefamandole, CTX; Cefotaxime, P; Penicillin, S; Streptomycin, AN; Amikacin, NB; Novobiocin, FOX; Cefoxitin, AMC; Amoxicillin/Clavulanic Acid, K; Kanamycin, CEC; Cefaclor.
